# Hamstring Architectural and Functional Adaptations Following Long vs. Short Muscle Length Eccentric Training

**DOI:** 10.3389/fphys.2016.00340

**Published:** 2016-08-03

**Authors:** Kenny Guex, Francis Degache, Cynthia Morisod, Matthieu Sailly, Gregoire P. Millet

**Affiliations:** ^1^Department of Physiotherapy, University of Health Sciences (Haute Ecole de Santé Vaud), University of Applied Sciences and Arts Western Switzerland (Haute École Spécialisée de Suisse Occidentale)Lausanne, Switzerland; ^2^Department of Physiology, Faculty of Biology and Medicine, Institute of Sport Sciences, University of LausanneLausanne, Switzerland; ^3^Centre Medical SynergieLausanne, Switzerland

**Keywords:** hamstring, eccentric, muscle length, fascicle length, pennation angle, optimum angle, strength

## Abstract

Most common preventive eccentric-based exercises, such as Nordic hamstring do not include any hip flexion. So, the elongation stress reached is lower than during the late swing phase of sprinting. The aim of this study was to assess the evolution of hamstring architectural (fascicle length and pennation angle) and functional (concentric and eccentric optimum angles and concentric and eccentric peak torques) parameters following a 3-week eccentric resistance program performed at long (LML) vs. short muscle length (SML). Both groups performed eight sessions of 3–5 × 8 slow maximal eccentric knee extensions on an isokinetic dynamometer: the SML group at 0° and the LML group at 80° of hip flexion. Architectural parameters were measured using ultrasound imaging and functional parameters using the isokinetic dynamometer. The fascicle length increased by 4.9% (*p* < 0.01, medium effect size) in the SML and by 9.3% (*p* < 0.001, large effect size) in the LML group. The pennation angle did not change (*p* = 0.83) in the SML and tended to decrease by 0.7° (*p* = 0.09, small effect size) in the LML group. The concentric optimum angle tended to decrease by 8.8° (*p* = 0.09, medium effect size) in the SML and by 17.3° (*p* < 0.01, large effect size) in the LML group. The eccentric optimum angle did not change (*p* = 0.19, small effect size) in the SML and tended to decrease by 10.7° (*p* = 0.06, medium effect size) in the LML group. The concentric peak torque did not change in the SML (*p* = 0.37) and the LML (*p* = 0.23) groups, whereas eccentric peak torque increased by 12.9% (*p* < 0.01, small effect size) and 17.9% (*p* < 0.001, small effect size) in the SML and the LML group, respectively. No group-by-time interaction was found for any parameters. A correlation was found between the training-induced change in fascicle length and the change in concentric optimum angle (*r* = −0.57, *p* < 0.01). These results suggest that performing eccentric exercises lead to several architectural and functional adaptations. However, further investigations are required to confirm the hypothesis that performing eccentric exercises at LML may lead to greater adaptations than a similar training performed at SML.

## Introduction

Hamstring strain injuries, and especially biceps femoris long head strain injuries, are among the most frequent injuries in sports requiring high-speed running, such as football, rugby, Australian football, Gaelic football, American football, or track and field (Brooks et al., 2005; Ekstrand et al., 2011; Elliott et al., 2011; Alonso et al., 2012; Murphy et al., 2012; Orchard et al., 2013). The long head of the biceps femoris has higher injury susceptibility at faster sprinting speed, since peak local fiber strain, fiber strain non-uniformity, and the amount of muscle undergoing larger strains are increased at faster speeds (Fiorentino et al., 2014). The late swing phase of sprinting is believed to be the main period of susceptibility to hamstring strain injuries (Chumanov et al., 2012). During this phase, the hamstring are undergoing an eccentric contraction to decelerate the knee extension, while the hip is flexed. Combined with the knee extension movement, this hip flexion position induces a substantial elongation stress on the muscle-tendon unit of the bi-articular hamstrings (elongation stress = hip flexion angle—knee flexion angle; Schache et al., 2012; Guex and Millet, 2013).

The evidence from randomized controlled trials are inconclusive to draw conclusions on the effectiveness of interventions used to prevent hamstring injuries (Goldman and Jones, 2010). The complex nature of hamstring strain injuries made that no one-single approach can be considered the gold standard for prevention (Opar et al., 2012). Nonetheless, eccentric-based intervention has been shown to be a promising method to reduce the risk of hamstring strain injuries (Askling et al., 2003; Brooks et al., 2006; Gabbe et al., 2006; Arnason et al., 2008; Petersen et al., 2011; Nichols, 2013). This may be explained by the increase in eccentric strength and by the observed shift of the optimum angle (i.e., the angle at which peak torque occurs) in the direction of longer muscle length following an eccentric strength program (Brockett et al., 2001; Askling et al., 2003; Mjolsnes et al., 2004; Clark et al., 2005; Kilgallon et al., 2007; Potier et al., 2009; Reeves et al., 2009; Brughelli et al., 2010; Martínez-Ruiz et al., 2014; Guex et al., 2016; Timmins et al., 2016a). Along with the increase in strength, eccentric-based interventions induce neuro-muscular adaptations, including improvement in the neural factors, muscle hypertrophy and an increase in fascicle pennation angle, suggesting an addition of sarcomeres in parallel (Moritani and deVries, 1979; Kawakami et al., 1993; Aagaard et al., 2001; Reeves et al., 2004; Blazevich et al., 2007; Seynnes et al., 2007). Whilst there is a general consensus that increases in pennation are driven by hypertrophy, a lack of relationship between changes in muscle size and changes in pennation can be observed. In line, with this unclear relationship, two studies have shown no modification or a slight decrease in pennation angle of the biceps femoris following eccentric training (Potier et al., 2009; Timmins et al., 2016a). The shift of the optimum angle, is often attributed to an increase in fascicle length, suggesting an addition of sarcomeres in series within the muscle, which enables operating over a greater range of motion without overstretch (Blazevich et al., 2007; Seynnes et al., 2007; Potier et al., 2009; Reeves et al., 2009). But, to date no research has examined the direct effect of a change in fascicle lengths on the risk of injury (Timmins et al., 2016b). Other contraction modes, such as the isometric, can also cause angle-specific adaptations and shift in optimum angle (Kitai and Sale, 1989). This may be explained by the fact that other mechanisms, such as neural factors may also influence the force-length relationship. Indeed, an improvement in rate of force development would also contribute to a shift in OA in the direction of longer muscle length (Aagaard et al., 2002). Moreover, recent detailed evidence suggest a possible role of region-specific muscle hypertrophy in addition to a neural mechanism (Noorkoiv et al., 2014).

In order to optimize the hamstring strength exercises in a perspective of injuries prevention, a conceptual framework based on the biomechanical parameters of sprinting has been recently proposed (Guex and Millet, 2013). It is suggested to use eccentric contractions performed at a slow to moderate angular velocity with a movement focused at the knee joint, while the hip is kept in a large flexion position in order to reach an elongation stress of the hamstrings greater than in the late swing phase (Guex and Millet, 2013). The most common eccentric-based interventions, which were shown to be efficient to reduce hamstring strain injuries used either the Nordic hamstring exercise (Brooks et al., 2006; Gabbe et al., 2006; Arnason et al., 2008; Petersen et al., 2011; Nichols, 2013), or the yo-yo hamstring curl exercise (Askling et al., 2003). These two exercises involve eccentric contractions performed at a slow to moderate angular velocity with a movement focused at the knee joint. However, they include only minimal hip flexion. Then, the elongation stress reached at the end of these two movements is close to 0 (< 30° of hip flexion—0° of knee flexion), while it is widely positive during the late swing phase (>70° of hip flexion— < 30° of knee flexion; Novacheck, 1998; Thelen et al., 2005). The elongation stress seems a relevant injury risk factor: the magnitude of musculotendinous lengthening occurring during repeated eccentric contractions was related to the severity of the subsequent muscle damage (Lieber and Friden, 1993). Moreover, some evidence have reported that the training range of motion (i.e., muscle excursion range during loading) could be the dominant stimulus for adaptations in fascicle length (Blazevich et al., 2007).

To our knowledge, it is unknown how the hamstring would adapt following an eccentric resistance training performed at long (i.e., positive elongation stress) vs. short (i.e., negative elongation stress) muscle length. Then, the aim of this study was to assess the change in: (1) hamstring architectural parameters (fascicle length and pennation angle of the long head of the biceps femoris); (2) hamstring functional parameters (concentric and eccentric optimum angles and concentric and eccentric peak torques) following a 3-week eccentric resistance program performed at long (LML) vs. short muscle length (SML). Since muscle architectural adaptations have been shown to rapidly occur (Blazevich et al., 2007; Seynnes et al., 2007; Timmins et al., 2016a), it was hypothesized that the proposed eccentric training would increase the fascicle length, the pennation angle and the strength, and would decrease the optimum angle in both groups. Furthermore, it was hypothesized that architectural and functional adaptations would be greater following eccentric training performed at LML vs. SML.

## Materials and methods

### Subjects

Twenty-two subjects were recruited voluntarily to the study and randomly allocated into two equal sized groups: the short (SML) and long (LML) muscle length groups. Table 1 summarizes the characteristics of the two groups, which were similar in age (*p* = 0.55), height (*p* = 0.47), and body mass (*p* = 0.72). The subjects performed only recreational physical activity. They were excluded if they reported traumatological disorders, history of hip or knee pathology or dysfunction. Prior to the beginning of the study, the subjects signed an informed consent after explanation of the study protocol, data collection procedures, significance of the study objectives, benefits, and risks of the investigation. Ethical approval for the project was obtained from the local committee on human research (Commission cantonale d'éthique de la recherche sur l'être humain, CCER-VD, Agreement 181/15, Lausanne, Switzerland).

**Table 1:** **Subject's characteristics (mean ± *SD*)**.

	**SML group (*n* = 11)**	**LML group (*n* = 11)**
Female/male ratio	6/5	6/5
Age, years	27.3 ± 3.9	28.4 ± 4.5
Height, cm	173.5 ± 10.8	170.7 ± 5.9
Weight, kg	66.0 ± 13.6	64.0 ± 12.7

SML, short muscle length; LML, long muscle length.

### Experimental design

For 3-weeks, the subjects completed eight hamstring eccentric sessions on an isokinetic dynamometer (Biodex System 2, Biodex Medical Systems, Shirley, New York, USA). In order to modify the hamstring muscle length, the SML group performed all training sessions in a supine position and the LML in a seated position. Ultrasound and isokinetic measurements were performed before the first training session and 4 days after the last training session in order to assess the evolution of: (1) hamstring architectural parameters: fascicle length (FL) and pennation angle (PA) of the long head of the biceps femoris; (2) hamstring functional parameters: optimum angle in concentric at 60°/s (ConOA), optimum angle in eccentric at 30°/s (EccOA), concentric peak torque at 60°/s (ConPT), and eccentric peak torque at 30°/s (EccPT).

### Eccentric resistance training

Prior to each session, the subjects performed a 10-min warm-up on a cycling ergometer (60 rpm, 80 watts). They were then placed on the isokinetic dynamometer. Stabilization straps were positioned across their chest, pelvis, and thigh. The lever arm shin-pad was positioned just proximal to the lateral malleolus. For practical reasons, the subjects were all trained and tested on their right limb.

The eccentric resistance training consisted in eight sessions performed during 3-weeks: two sessions per week the 1st week and three sessions in second and 3rd weeks. Each session consisted of three (weeks 1), four (week 2), or five (week 3) sets of eight maximal eccentric knee extensions performed at 30°/s on the isokinetic dynamometer. A 3-min rest period was allocated between each set. For each repetition, the knee range of motion was fixed at 110° (between 110 and 0° of knee flexion, 0° corresponding to full extension).

**Figure 1:**
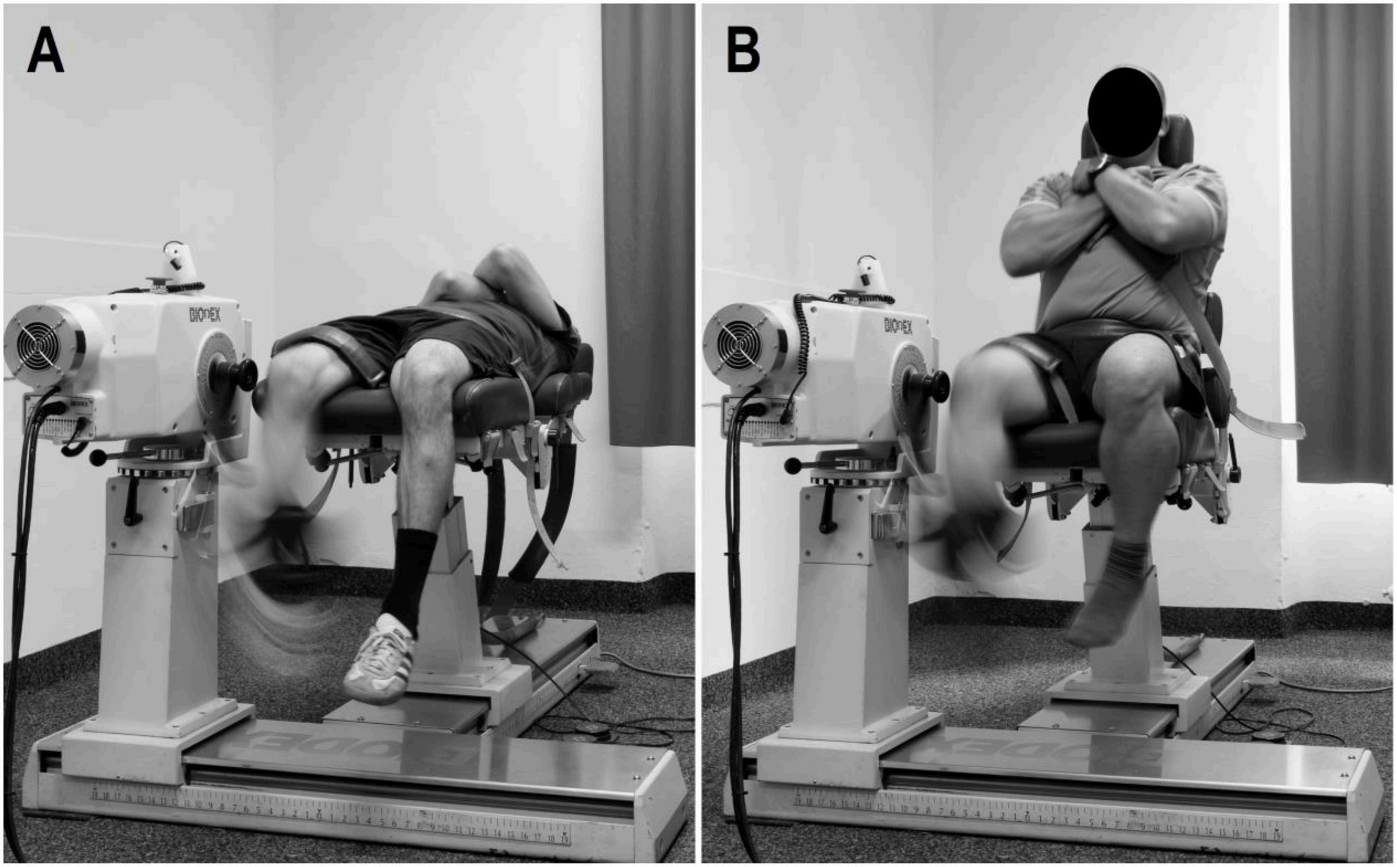
**Hamstring eccentric resistance training at (A) short and (B) long muscle length**.

Both SML and LML groups followed the same training program. However, in order to modify the hamstring muscle length, the SML group performed the resistance training at 0° of hip flexion (i.e., supine position; Figure 1A) and the LML group at 80° of hip flexion (i.e., seated position; Figure 1B). Thus, during each repetition, the elongation stress moved from −110 to 0 in the SML group, while it moved from −30 to 80 in the LML group (Guex and Millet, 2013).

### Architectural adaptations assessment

Architectural parameters were assessed using ultrasound imaging, which was shown to be a reliable method for measuring FL and PA of the long head of the biceps femoris (Chleboun et al., 2001). Subjects were positioned in a prone position with their knee fully extended and their muscles relaxed. The probe (42 mm linear array transducer, 10 MHz wave frequency) of the ultrasonic instrument (SSD-2000, ALOKA, Tokyo, Japan) was placed directly on the skin above the middle-belly of the long head of the biceps femoris with transmission gel to obtain acoustic coupling. The probe was oriented parallel to the muscle fascicles and perpendicular to the skin. Once the probe was appropriately placed, its position was marked on the skin in order to replace it in the same position after the 3-weeks training program. After each training session, the mark was controlled and redrawn if necessary.

**Figure 2:**
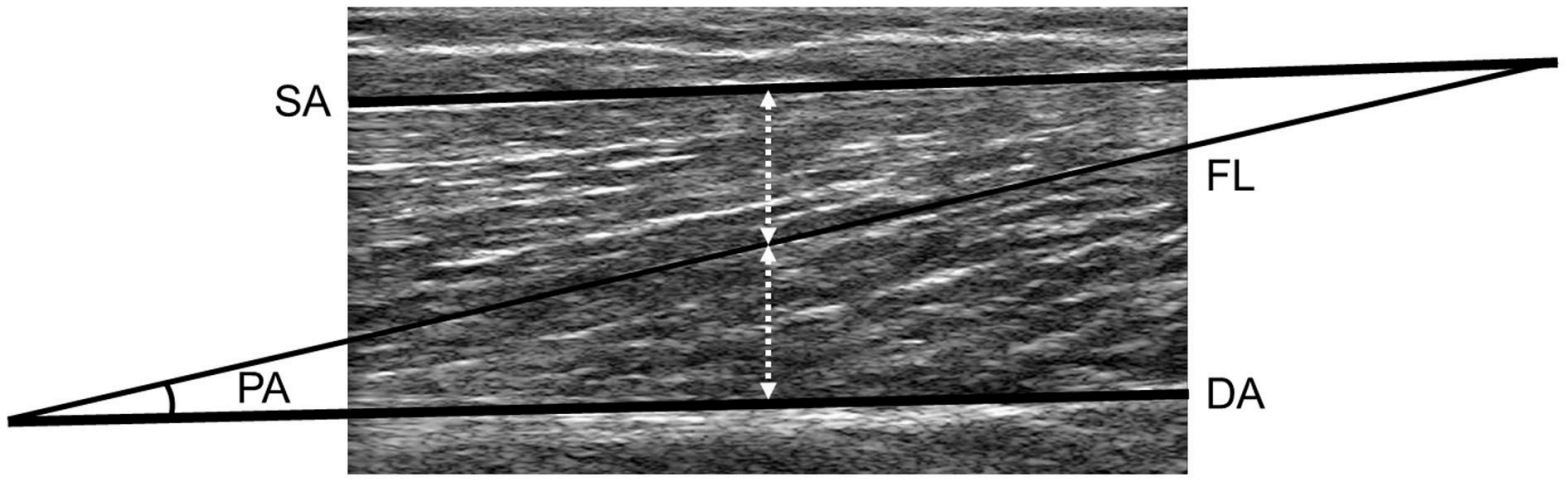
**Ultrasound image of the long head of the biceps femoris**. SA and DA lines represent the superficial and deep aponeurosis, respectively. FL corresponds to the fascicle length of the muscle fascicle, which joins SA and DA lines and crosses the midpoint between the two aponeurosis in the center of the image. PA represents the pennation angle, which was calculated as the angle between FL and DA.

FL was measured by manually outlining visible parts of the muscle fascicle, which crossed the midpoint between the superficial and deep aponeurosis in the center of the ultrasound image. The length of the missing portions was estimated by measuring the linear distances from the identifiable ends of a fascicle to the intersection between the line drawn from the fascicle and the line drawn from the deep to the superficial aponeurosis (Blazevich et al., 2007). The angle between the line marking the deep aponeurosis and the outlined fascicle corresponded to the PA (Figure 2). Test-retest of architectural parameters assessment on three consecutive days in 10 control subjects indicated FL and PA measurements had a coefficient of variation (CV) of 2.1% (~1.8 mm) and 2.3% (~0.32°), respectively.

### Functional adaptations assessment

To assess the functional parameters, maximal concentric and eccentric strength of the right hamstring was measured using the isokinetic dynamometer. Pre- and post-training assessments took place at the same time of the day. The dynamometer was calibrated according to the manufacturer's recommendations and following the instructions for optimal reproducibility. Prior to the testing procedures, the subjects performed a similar warm-up as described in the eccentric resistance training part. They were then correctly placed on the dynamometer. The hip flexion angle was held at 80° (i.e., seated position) and the knee range of motion was similar as described in the eccentric resistance training part. The 80° hip flexion position was chosen in order to test the hamstring of each subject at a sprint specific angle (Guex et al., 2012; Guex and Millet, 2013). Before testing, gravity correction was obtained by measuring the torque exerted on the lever arm shin-pad with the knee in extension in a relaxed state. Prior to the pre-training assessment, familiarization sets were performed, consisting in 10 progressive concentric knee flexions at 60°/s followed by 10 progressive eccentric knee extensions at 30°/s with a 1-min rest period between both sets.

The isokinetic test consisted of a set of six maximal concentric knee flexions at 60°/s followed by six maximal eccentric knee extensions at 30°/s with a 3-min rest period between both sets. OA and PT were determined by fitting a 4th order polynomial curve to the raw data of the best repetition. OA corresponded to the angle at which the PT was reached. In addition, to complete the analysis on the torque-angle relationship, mean concentric and eccentric torques between 10°–20°, 20°–30°, 30°–40°, 40°–50°, 50°–60°, 60°–70°, 70°–80°, 80°–90°, and 90°–100° of knee flexion were recorded at pre- and post-training assessments.

### Statistical analysis

Data are expressed as mean ± standard deviation (*SD*). They were screened for a normal distribution using Shapiro-Wilk normality tests. To assess assumptions of variance, Mauchly's test of sphericity was performed. In order to observe the evolution of architectural and functional parameters following the eccentric resistance training, two-way (group × time) analysis of variance (ANOVA) with repeated measures were used. Tukey *post-hoc* tests were used to localize the differences between means. The importance of the differences found between pre- and post-training assessment were assessed through the effect size and Cohen's *d* coefficient (Cohen, 1988), interpreted as follows: small difference: 0.15 ≤ *d* < 0.40, medium difference: 0.40 ≤ *d* < 0.75, large difference: 0.75 ≤ *d* < 1.10, and very large difference: *d*≥1.10. Finally, the relationship between changes in architectural and functional adaptations were examined using a Pearson correlation coefficients, interpreted as follows: weak: 0.20 ≤ *r* < 0.40, moderate: 0.40 ≤ *r* < 0.60, good: 0.60 ≤ *r* < 0.80 and excellent: 0.80 ≤ *r* ≤ 1.00. For all statistical analysis, significance was set at *p* < 0.05. Statistical analysis were performed with SigmaPlot 12.5 (Systat Software Inc., San Jose, CA).

## Results

The values of architectural and functional parameters before and after the eccentric resistance training are presented in Table 2. At pre-, no difference in FL (*p* = 0.56), PA (*p* = 0.72), ConOA (*p* = 0.83), EccOA (*p* = 0.94), ConPT (*p* = 0.58) and EccPT (*p* = 0.47) was observed between the SML and the LML groups.

**Table 2:** **Hamstring architectural and functional parameters (mean ± *SD*) before (Pre-) and after (Post-) the eccentric resistance training performed at short vs. long muscle length**.

	**SML group (n = 11)**				**LML group (n = 11)**			
	**Pre-**	**Post-**	**Net change [95% CI]**	**Effect size**	**Pre-**	**Post-**	**Net change [95% CI]**	**Effect size**
**ARCHITECTURAL PARAMETERS**								
FL, mm	84.1 ± 7.3	88.2 ± 7.9^**^	4.1 [2.5; 5.7]	0.57 (medium)	82.0 ± 9.3	89.4 ± 8.13^***^	7.4 [4.5; 10.2]	0.89 (large)
PA, °	15.0 ± 2.9	14.9 ± 2.2	−0.1 [0.7; −0.9]	0.04	14.6 ± 3.4	13.8 ± 3.0	−0.7 [0.1; −1.5]	0.24 (small)
**FUNCTIONAL PARAMETERS**								
ConOA, °	77.0 ± 16.5	68.3 ± 18.7	−8.8 [−1.6; −15.9]	0.52 (medium)	78.8 ± 14.8	61.5 ± 24.49^**^	−17.3 [−5.6; −29.0]	0.90 (large)
EccOA, °	40.0 ± 21.8	32.7 ± 25.1	−7.2 [0.9; −15.4]	0.32 (small)	40.6 ± 19.3	30.0 ± 17.2	−10.7 [1.8; −23.1]	0.61 (medium)
ConPT, Nm	47.4 ± 16.9	49.0 ± 16.1	1.7 [−1.5; 4.8]	0.11	43.1 ± 17.8	45.3 ± 19.9	2.3 [−1.7; 6.2]	0.13
EccPT, Nm	59.4 ± 22.9	65.5 ± 21.0^**^	6.1 [3.6; 8.7]	0.29 (small)	52.1 ± 23.9	60.1 ± 25.3^**^	8.0 [3.7; 12.3]	0.34 (small)

*SML, short muscle length; LML, long muscle length; CI, confidence interval; FL, fascicle length; PA, pennation angle; OA, optimum angle; Con, concentric; Ecc, eccentric; PT, peak torque*.

** p < 0.01 and

*** *p < 0.001 for differences with pre-training*.

### Architectural adaptations

Following the eccentric resistance training, the FL increased by 4.9% (*p* < 0.01, medium effect size) in the SML and by 9.3% (*p* < 0.001, large effect size) in the LML group. The PA did not change (*p* = 0.83) in the SML and tended to decrease by 0.7° (*p* = 0.09, small effect size) in the LML group. No group-by-time interaction was found for FL (*p* = 0.74) and PA (*p* = 0.39).

### Functional adaptations

The ConOA tended to decrease by 8.8° (*p* = 0.09, medium effect size) in the SML and by 17.3° (*p* < 0.01, large effect size) in the LML group. The EccOA did not change (*p* = 0.19, small effect size) in the SML and tended to decrease by 10.7° (*p* = 0.06, medium effect size) in the LML group. The ConPT did not change in the SML (*p* = 0.37) and the LML (*p* = 0.23) groups, whereas EccPT increased by 12.9% (*p* < 0.01, small effect size) and 17.9% (*p* < 0.001, small effect size) in the SML and the LML group, respectively. No group-by-time interaction was found for ConOA (*p* = 0.41), EccOA (*p* = 0.76), ConPT (*p* = 0.63), and EccPT (*p* = 0.59).

**Figure 3:**
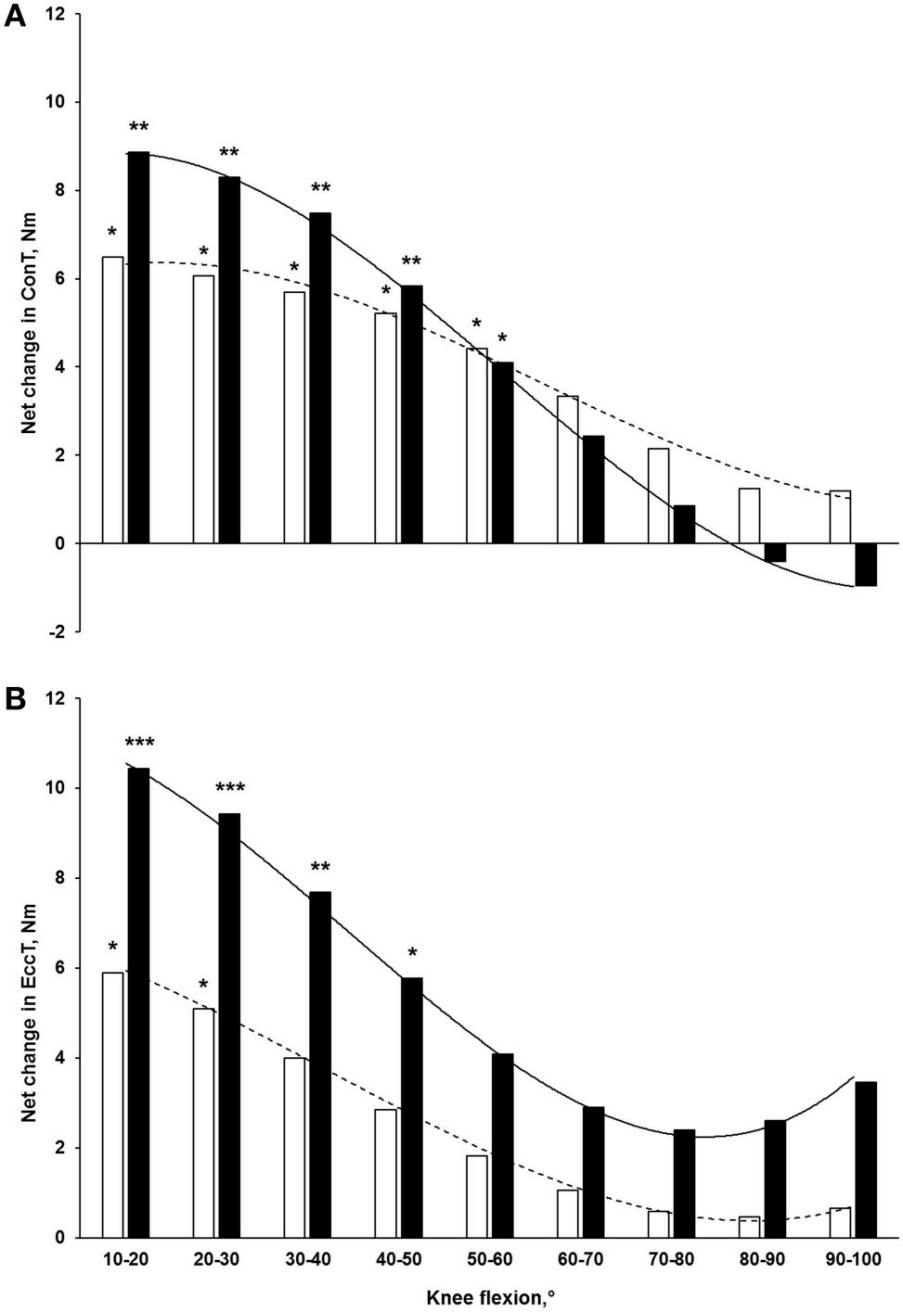
**Net changes in mean torques at different knee flexions in (A) concentric at 60°/s and (B) eccentric at 30°/s following a 3-week eccentric program**. Light and solid blocks represent the SML and LML groups, respectively. ^*^p < 0.05, ^**^p < 0.01, and ^***^p < 0.001 for differences with pre-training.

Following the 3-week eccentric program, the mean concentric torque at 60°/s significantly increased by 23.3% (*p* < 0.05, small effect size) and 42.0% (*p* < 0.01, medium effect size) between 10°–20°, by 17.1% (*p* < 0.05, small effect size) and 31.4% (*p* < 0.01, medium effect size) between 20°–30°, by 14.4% (*p* < 0.05, small effect size) and 24.1% (*p* < 0.01, small effect size) between 30°–40°, by 12.5% (*p* < 0.05, small effect size) and 16.9% (*p* < 0.01, small effect size) between 40°–50° and by 10.3% (*p* < 0.05, small effect size) and 11.1% (*p* < 0.05, small effect size) between 50°–60° in the SML and the LML group, respectively (Figure 3A). The mean eccentric torque at 30°/s significantly increased by 10.4% (*p* < 0.05, small effect size) and 22.6% (*p* < 0.001, medium effect size) between 10°–20°, by 8.8% (*p* < 0.05, small effect size) and 19.1% (*p* < 0.001, medium effect size) between 20°–30° in the SML and the LML group, respectively. Moreover, it only significantly increased in LML group by 15.4% (*p* < 0.01, small effect size) and 11.7% (*p* < 0.05, small effect size) between 30°–40° and 40°–50°, respectively (Figure 3B). At each knee angle, no group-by-time interaction was found for both concentric and eccentric mean torques.

### Relationship between architectural and functional adaptations

A moderate negative correlation was found between the training-induced change in FL and the change in ConOA (*r* = −0.57, *p* < 0.01).

## Discussion

The aim of this study was to assess the changes in hamstring architectural and functional parameters following a 3-week eccentric resistance training performed at LML vs. SML. In line with our hypothesis, the FL and the EccPT significantly increased in both groups, the ConOA significantly decreased in the LML group and tended to decrease in the SML group, while the EccOA tended to decreased in the LML group. However, two parameters did not significantly change from pre- to post-training assessment: the PA and the ConPT. Furthermore, it was hypothesized that architectural and functional adaptations would be greater in the LML group than in the SML group. It is interesting to observe that for the changes in FL, ConOA, EccOA, mean concentric torque between 10° and 20° and between 20° and 30° and mean eccentric torque between 10° and 20° and between 20° and 30°, the effect sizes were greater in the LML than in the SML group. However, no group-by-time interaction was found. Further investigations are then required to confirm the hypothesis that performing eccentric exercises at a long muscle length could lead to greater adaptations than exercises performed with lesser hamstring elongation stress.

The mean FL- and PA-values of the long head of the biceps femoris observed at pre-training assessment (~83 mm and ~15°, respectively) are in line with previous studies (~58–117 mm and ~13°–19°, respectively; Chleboun et al., 2001; Woodley and Mercer, 2005; Potier et al., 2009; Kellis et al., 2010; Timmins et al., 2016a), showing the robustness of the present measurements. Following the 3-week eccentric program performed at short and long muscle length, the FL significantly increased by ~5 and 9%, respectively. These modifications are considerably lower than the ~34% increase observed by Potier et al. (2009) following an 8-week eccentric program performed in a prone position on a hamstring leg curl machine (i.e., at SML). They are also lower than the 15% increase obtained by Timmins et al., after 14 and 21 days of eccentric training on an isokinetic dynamometer with a protocol comparable to our intervention at long muscle length (Timmins et al., 2016a). Our results are consistent with those of Seynnes et al., who have reported ~2, 6, and 10% increases in vastus lateralis FL after 10, 20, and 35 days of eccentric training at long muscle length, respectively, or with Blazevich et al., who have found a ~3% increase in vastus lateralis FL following a 10-week eccentric program performed at long muscle length (Blazevich et al., 2007; Seynnes et al., 2007). In this later study, the authors have found a ~6% increase in vastus lateralis FL following a similar 10-week program performed in concentric, suggesting that, beyond the contraction mode, it is the training range of motion (or muscle excursion range) that is paramount for fascicle length adaptation. To our knowledge, the present study is the first one attempting to test this hypothesis by comparing two similar training interventions performed at different muscle lengths. Although the effect size was greater following the intervention performed at long muscle length, there was no group-by-time interaction. To show a significant difference (*p* < 0.05) between the increase in FL obtained in the LML (7.4 mm) and SML (4.1 mm) groups with 80% power, the sample size should be of 28 subjects in each group. This is an important limitation of the present study.

Following the 3-week eccentric program, the PA did not change in the SML group and tended to slightly decrease in the LML group. One may assume that this later results are the consequence of the lack of hypertrophy due to the short intervention, since eccentric training has classically been associated to an increase in PA, suggesting an addition of sarcomeres in parallel (Kawakami et al., 1993; Aagaard et al., 2001; Blazevich et al., 2007; Seynnes et al., 2007). However, Timmins et al. found a significant slight decrease in PA after only 14 days of hamstring eccentric training (Timmins et al., 2016a), and Potier et al., as in the present the study, reported a slight but not significant decrease in PA after their eccentric intervention on the hamstring (Potier et al., 2009). Potier et al. stated that any change in PA may be muscle specific, and that significant changes may have been observed in other knee flexor muscles, such as the semitendinosus.

After the intervention performed at long muscle length, the ConOA shifted by 17° in the direction of longer muscle length, while the EccOA tended to shift by 11°. At SML, only the ConOA tended to shift by 9°. These shifts are in line with previous studies on the hamstring (4°–21°; Clark et al., 2005; Kilgallon et al., 2007; Brughelli et al., 2010; Martínez-Ruiz et al., 2014). A decrease in OA is classically attributed to an increase in fascicle length, suggesting an addition of sarcomeres in series within the muscle (Blazevich et al., 2007; Seynnes et al., 2007; Potier et al., 2009; Reeves et al., 2009). Interestingly, this hypothesis is supported by the correlation found in the present study between the increase in FL and the shift in ConOA in the direction of longer muscle length, suggesting that the increase in fascicle length allows the muscle to operate effectively over a greater range of motion. However, one must be cautious when interpreting the relation between architectural and functional parameters, since no correlation was found between the increase in FL and the shift in EccOA (*r* = −0.17, *p* = 0.46). This may be explained by the fact that other factors, such as neural mechanism and/or region-specific muscle hypertrophy may also have influenced the force-length relationship. It is likely that the shift in OA is a multifactorial event. Nonetheless, the data presented in Figure 3 highlights the effect of the eccentric training on hamstring function: both concentric and eccentric torques preferentially increased at long muscle length (i.e., when the knee is in a low flexion position) with a greater training-induced adaptation in the LML group. This may be particularly relevant for injuries prevention in sprinters, since the late swing phase of sprinting has been shown to be the main period of susceptibility to hamstring strain injuries: during this phase, the hamstring are stretched by ~10% beyond their upright length (Chumanov et al., 2012; Schache et al., 2012). Thus, an eccentric intervention would help the hamstring to work more efficiently without overstretch during the whole sprinting cycle.

In both groups, the EccPT was significantly increased by more than 10% following the intervention, while the ConPT did not change. This large increase in eccentric strength demonstrates the efficiency of the present protocol. As discussed above, the PA did not increase in both groups, which could suggests a lack of hypertrophy. Then, the present results suggest that the increase in eccentric strength could be attributed to an improvement in the neural factors or in excitation–contraction coupling (Moritani and deVries, 1979; Warren et al., 2001; Reeves et al., 2004). However, an assessment of the electromyographic activity of the biceps femoris long head would have been necessary to confirm this hypothesis. The lack of improvement in ConPT after the isokinetic eccentric program is in line with previous studies and confirms the concept of mode specificity in isokinetic training (Tomberlin et al., 1991; Seger et al., 1998). Finally, one cannot rule out that the seated position allowed slightly greater torques in training (Guex et al., 2012). Then the small, non-significant additional changes seen in LML group might be associated with the greater training load, rather than the muscle length used in training.

The present study has some limitations. First, the proposed intervention consisted in only eight sessions over 3-weeks. Further, studies are required to investigate if larger differences between the LML and SML groups would occur with longer training period. While PA would probably have increased due to an increase in the amount of contractile tissue, one may though that FL would not have increased more, since it was shown that FL increase occurs mainly within the 1st weeks of training (Blazevich et al., 2007; Timmins et al., 2016a). Second, the LML group performed the resistance training at 80° of hip flexion. Thus, during each repetition, the elongation stress moved from −30 to 80, which is greater than the SML group (−110 to 0). However, one may think that the elongation stress reached in the LML group could not be enough to generate an hamstring overstretch in all subjects, especially in the more flexible ones. In future investigations, it could be relevant to individualize the range of motion of the LML group in regards to the flexibility of each subject to ensure a sufficient musculotendinous elongation stress. Another limitation of the present investigation concerns the assessment of the FL. Indeed, the length of the missing portions was estimated, which implies an important extrapolation. However, the reliability testing indicated FL measurements had a CV of 2.1% (~1.8 mm), which is ~2 and 4 times lower than the observed FL increase in SML and LML groups, respectively. Moreover, as previously mentioned, the FL-values of the present investigation are in line with those reported in previous studies (Chleboun et al., 2001; Woodley and Mercer, 2005; Potier et al., 2009; Kellis et al., 2010; Timmins et al., 2016a). Another potential limitation, is that no control group was included in the present study design. Therefore, one may not exclude that part of the observed adaptations could be due to non-controlled factors, even if the protocol (e.g., training loads, testing procedure, …) was perfectly controlled for each subject. Future controlled studies, are then required to reinforce the present findings. Finally, only the long head of the biceps femoris was analyzed. It is possible that the other knee flexor muscles would have responded differently to the present training protocol.

In conclusion, this study, which was the first one to investigate the influence of muscle length during eccentric training on hamstring architectural and functional parameters, reported eccentric strength, fascicle length, concentric, and eccentric optimum angles increased following eccentric intervention in both positions with no group-by-time interaction. However, fascicle length, concentric, and eccentric optimum angles increased with larger effect size following eccentric training at long than at SML. Further investigations, such as randomized controlled trials with larger sample size are required to confirm the hypothesis that performing eccentric exercises with a large elongation stress may lead to greater architectural and functional adaptations than a similar training performed at SML.

## Author contributions

Conceived and designed the experiments: KG, FD, MS, and GM. Performed experiments: KG, FD, and CM. Analyzed data: KG and CM. Interpreted results of research: KG, FD, MS, and GM. Drafted manuscript and prepared tables/figures: KG, FD, and CM. Edited, critically revised paper and approved final version of manuscript: KG, FD, CM, MS, and GM. All authors have agreed to be accountable for all aspects of the work related to its accuracy and integrity.

### Conflict of interest statement

The authors declare that the research was conducted in the absence of any commercial or financial relationships that could be construed as a potential conflict of interest. The reviewer AN and handling Editor declared their shared affiliation, and the handling Editor states that the process nevertheless met the standards of a fair and objective review.
